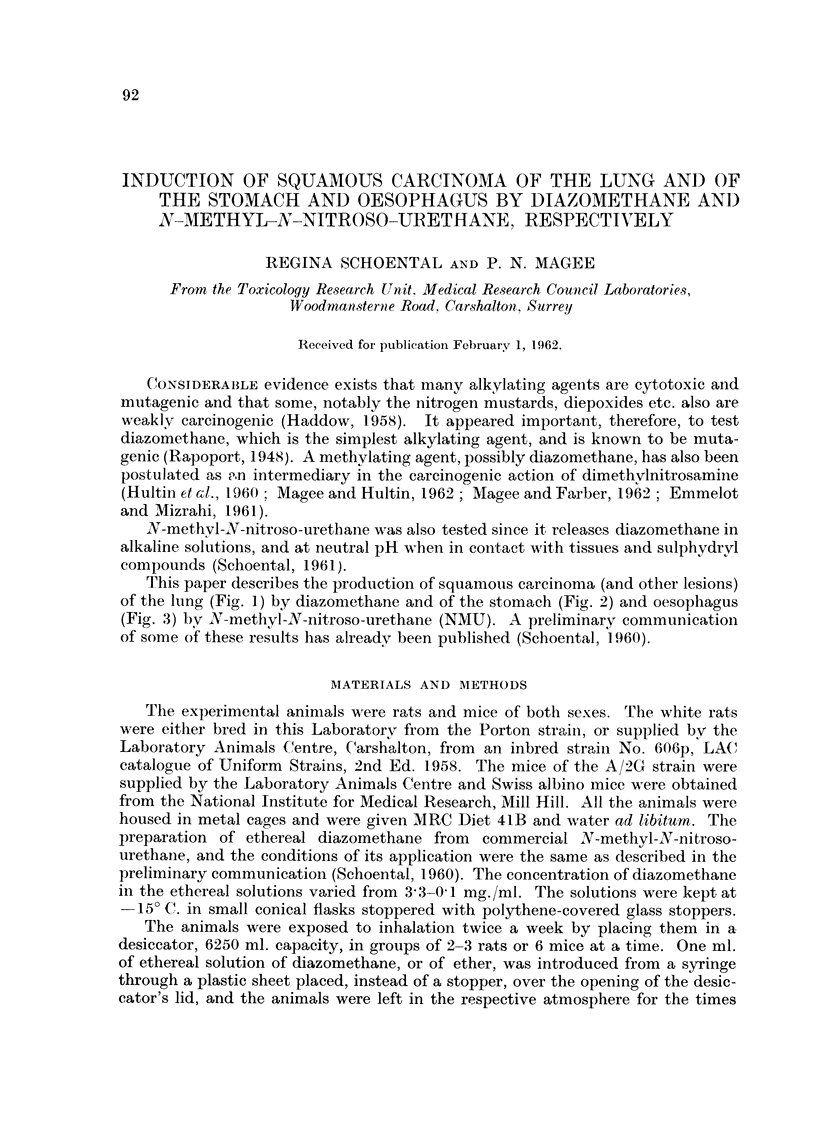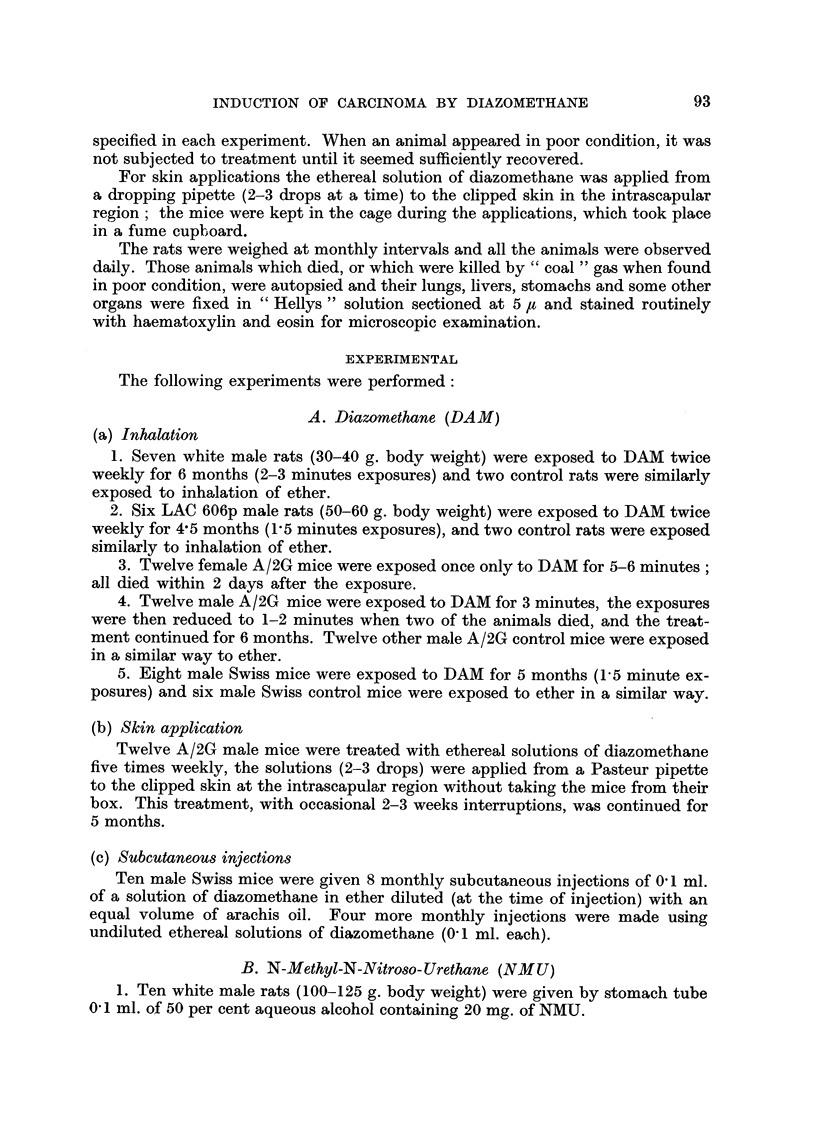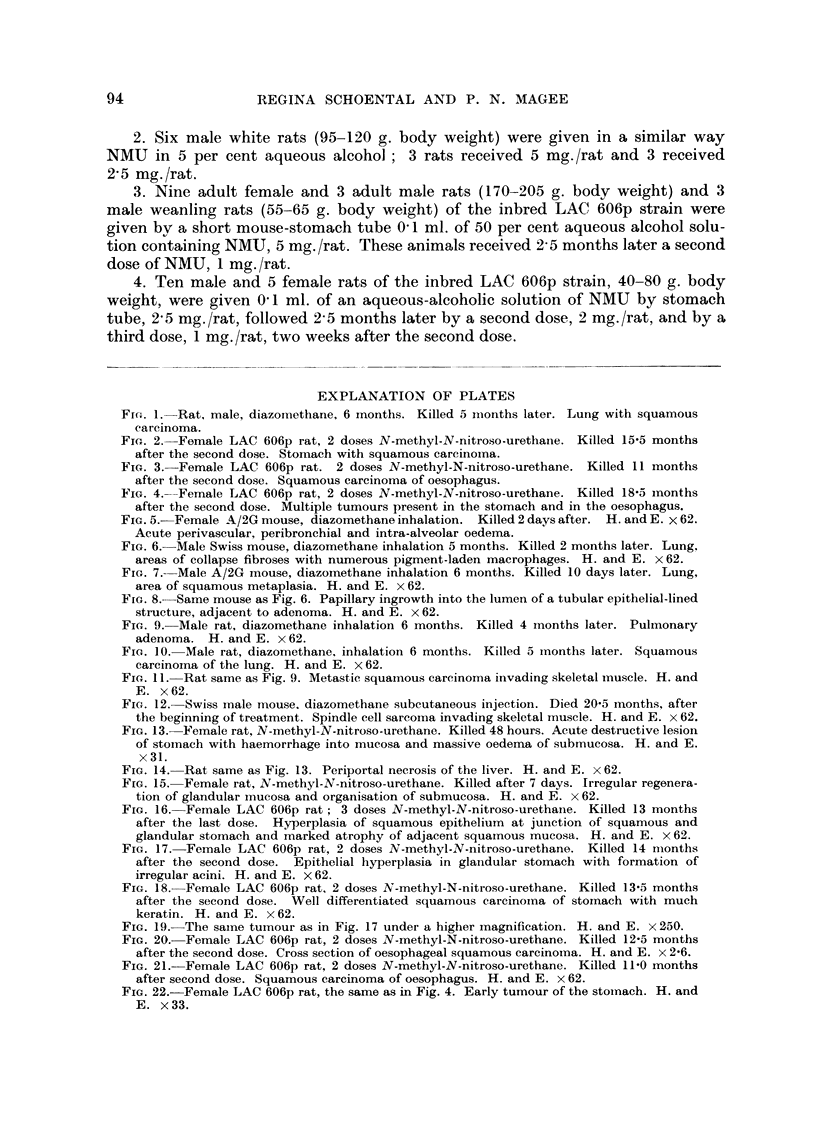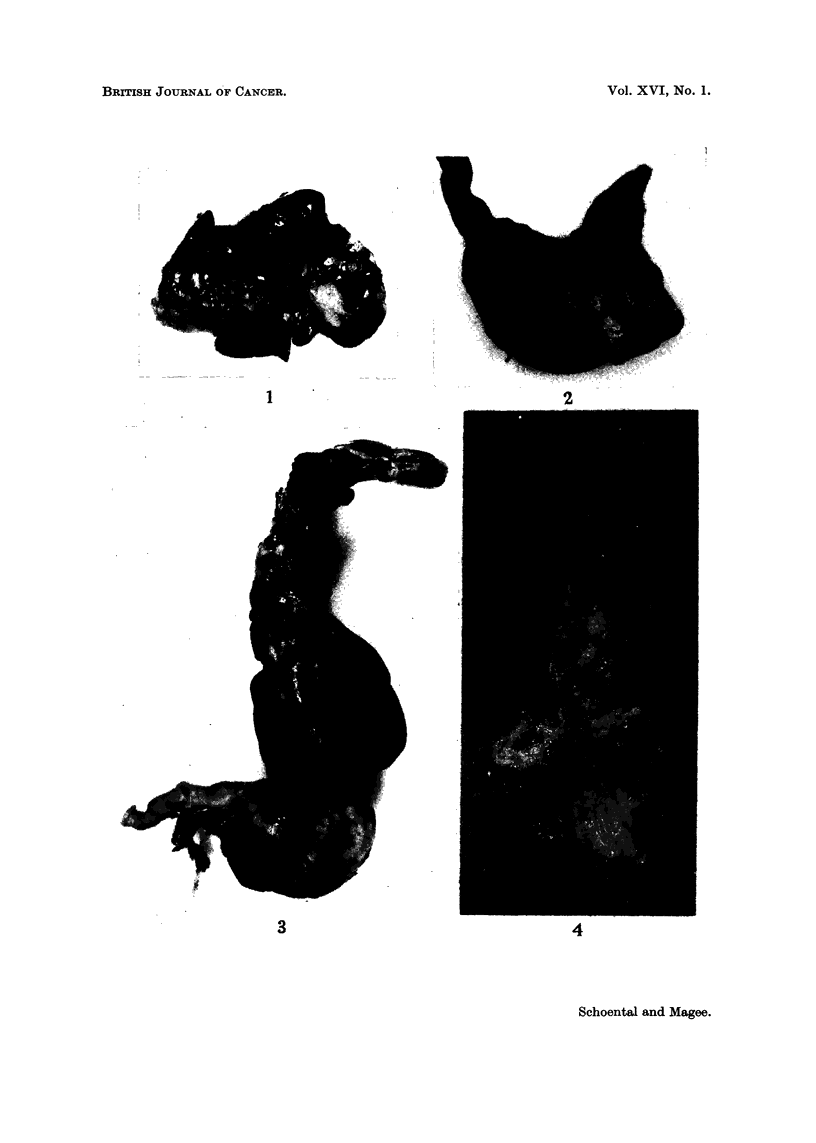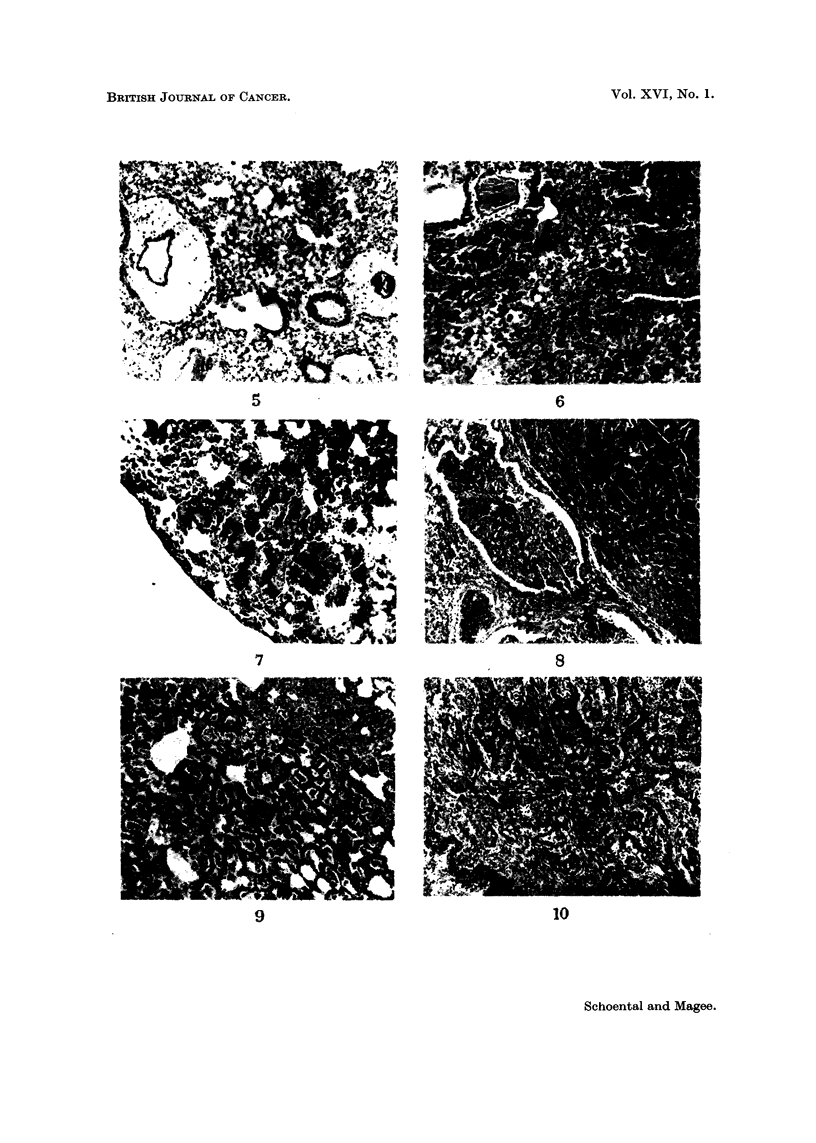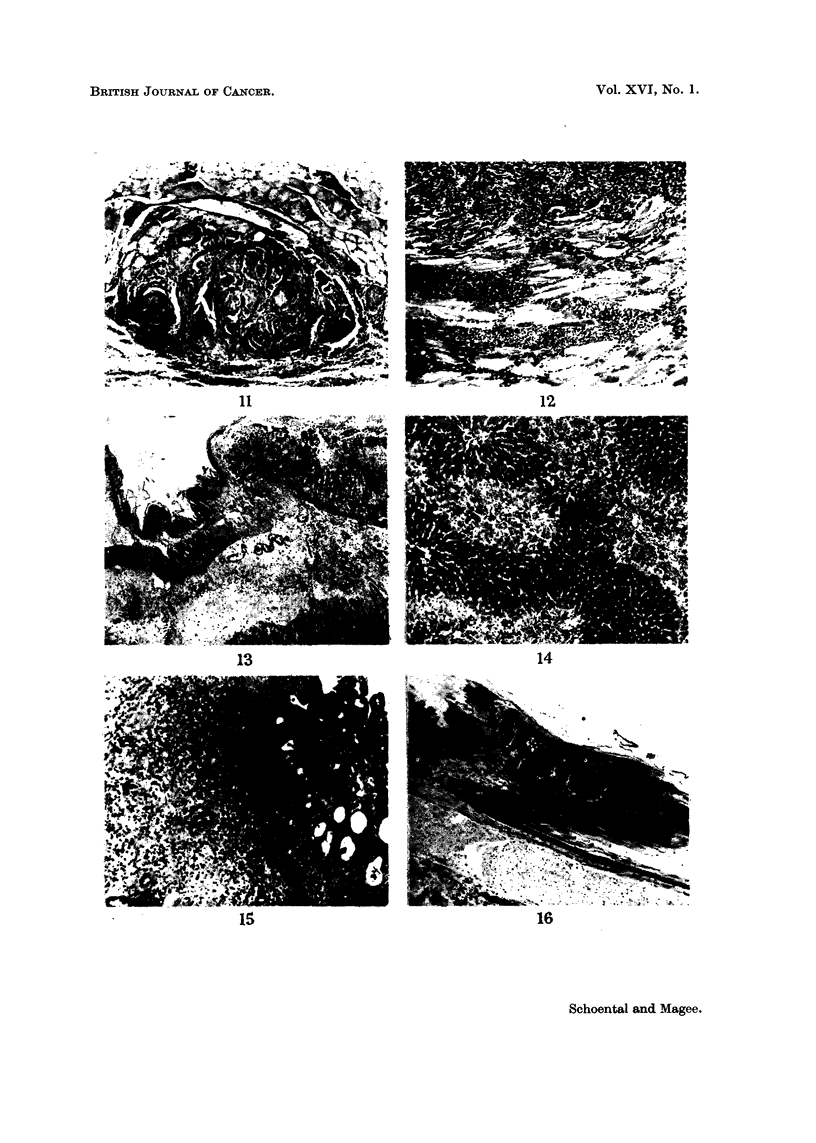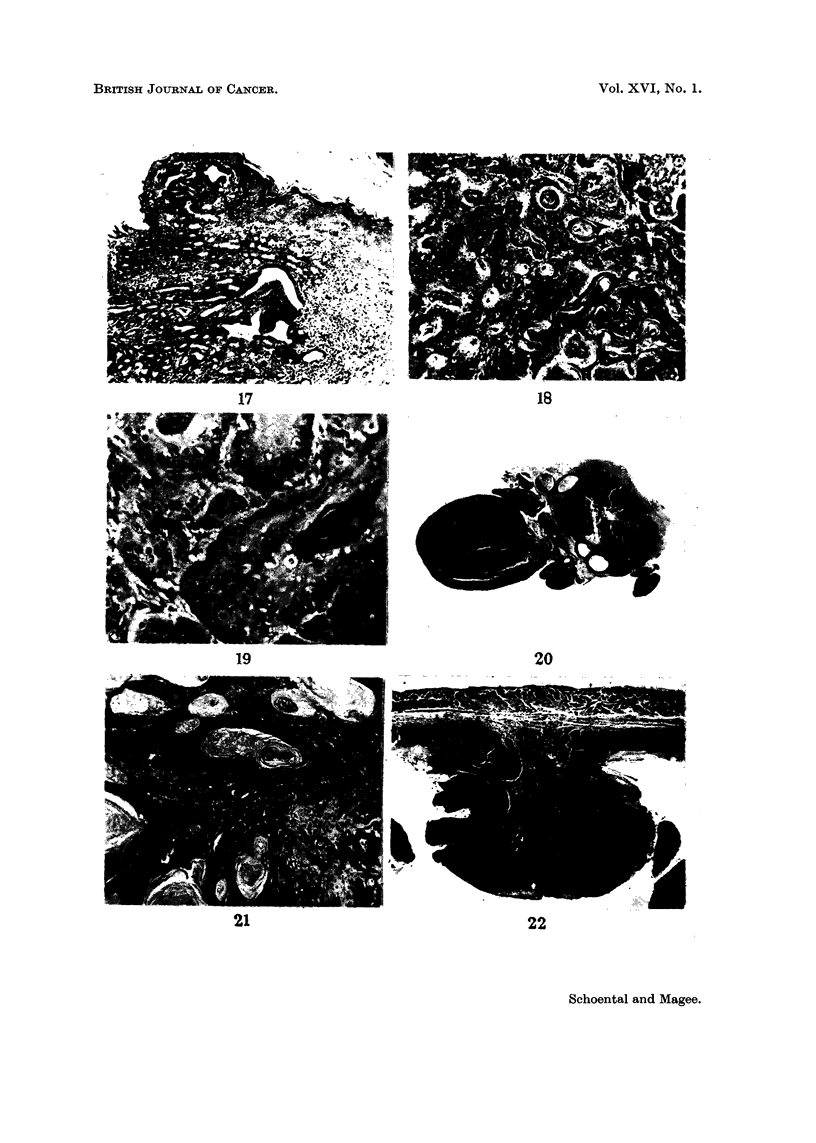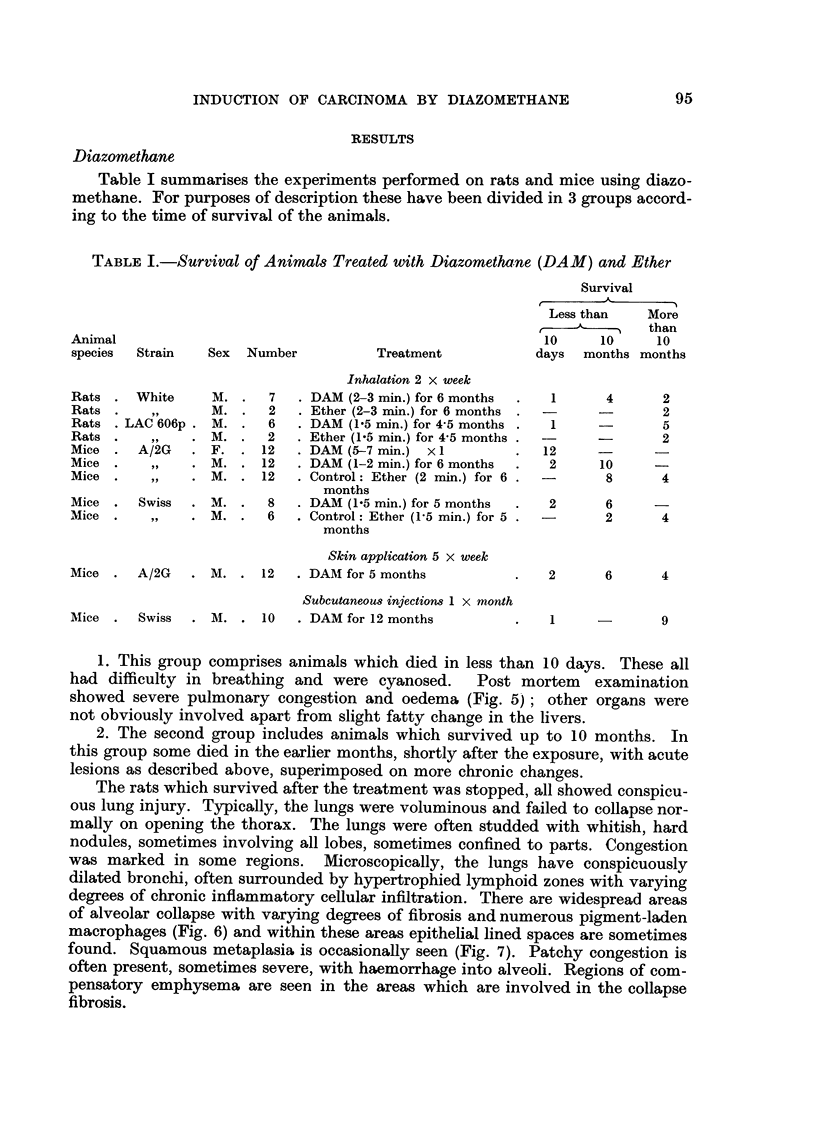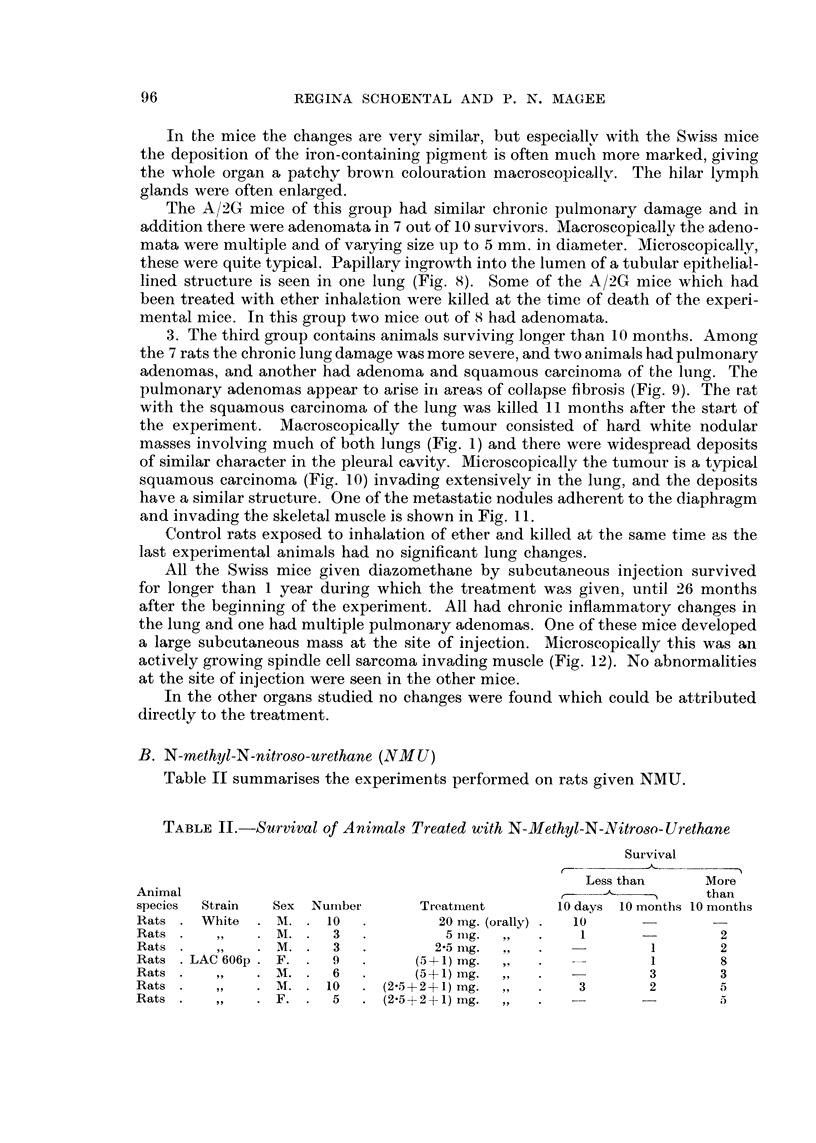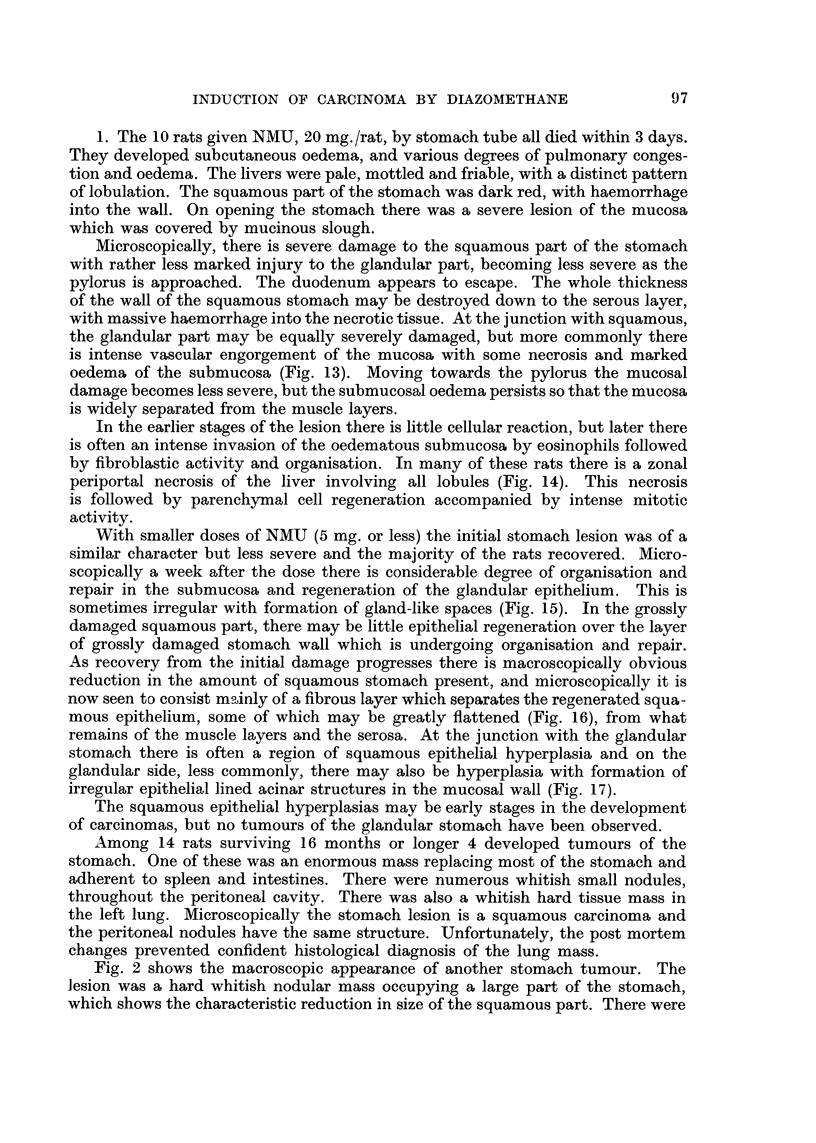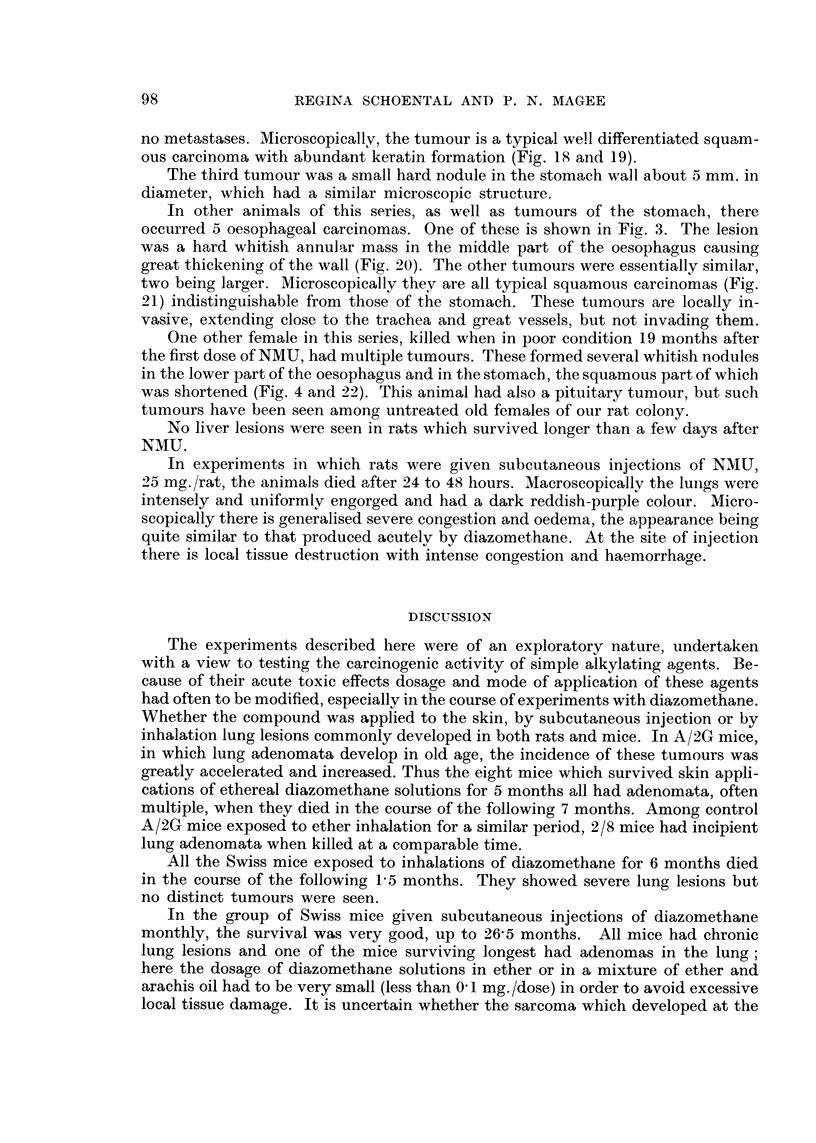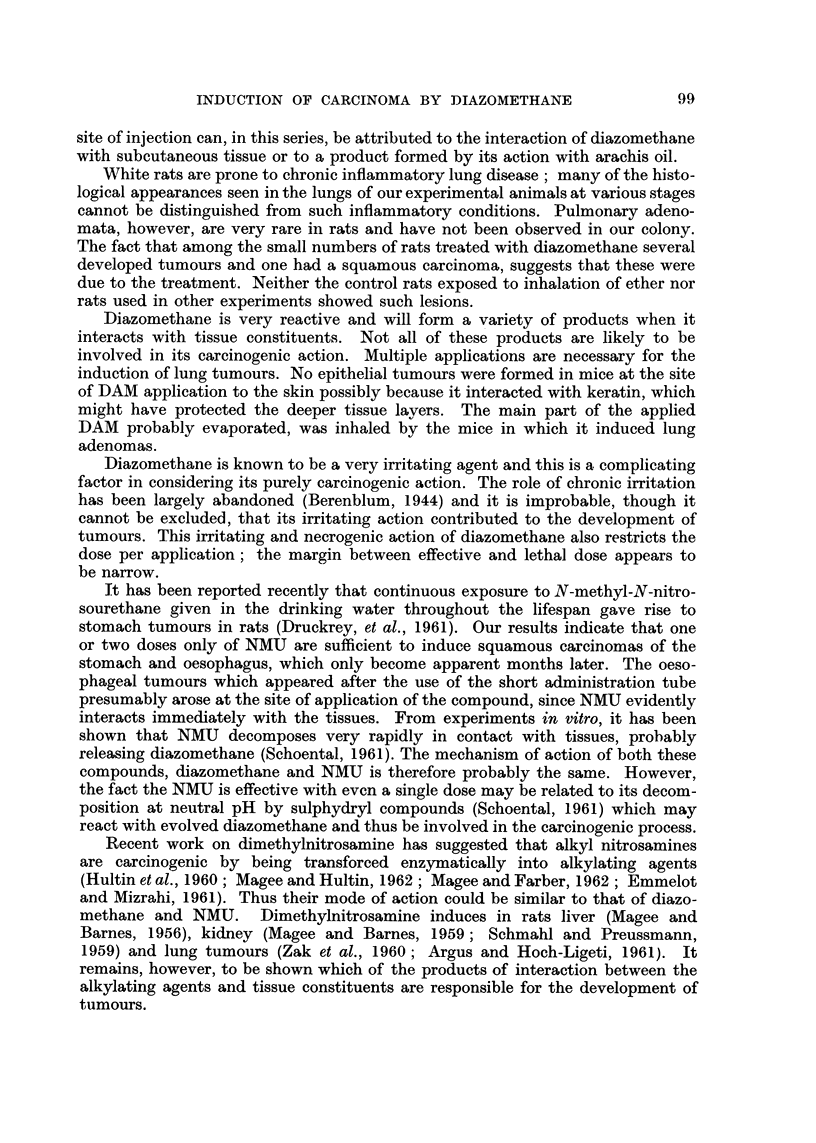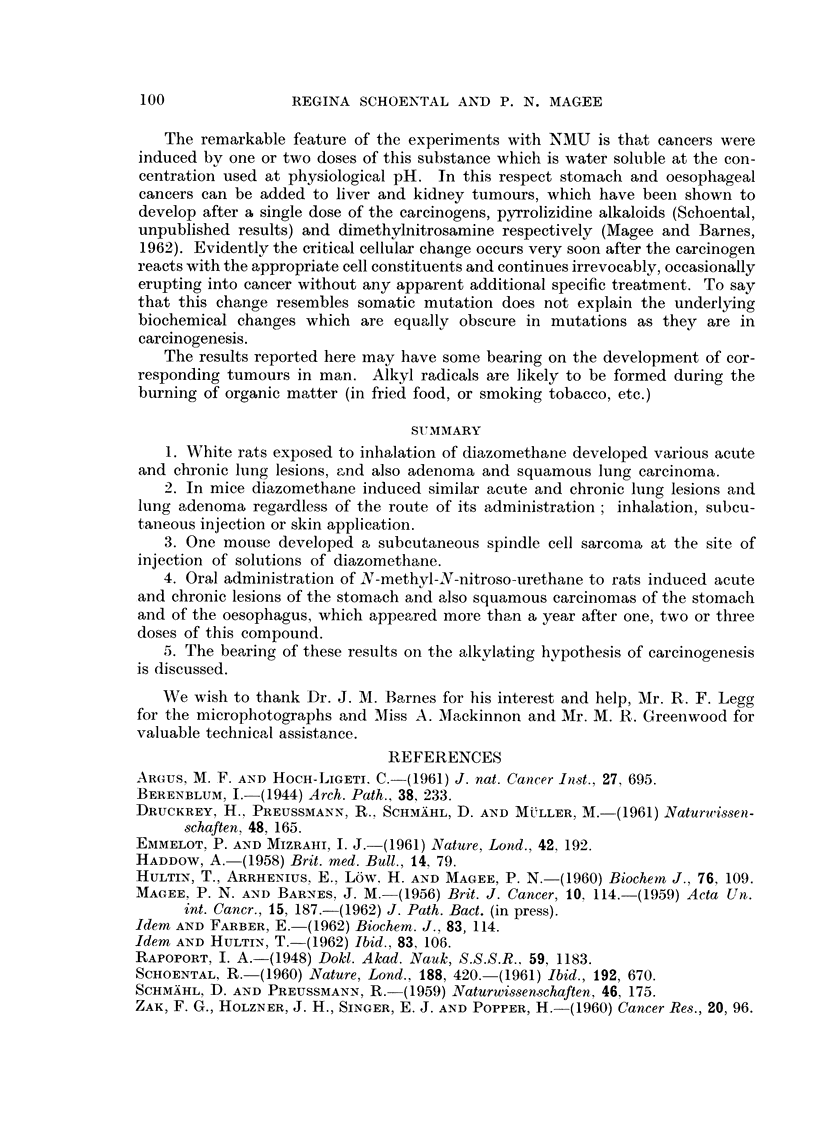# Induction of Squamous Carcinoma of the Lung and of the Stomach and Oesophagus by Diazomethane and N-Methyl-N-Nitroso-Urethane, Respectively

**DOI:** 10.1038/bjc.1962.9

**Published:** 1962-03

**Authors:** Regina Schoental, P. N. Magee

## Abstract

**Images:**


### INDUCTION OF SQUAMOUS CARCINOMA OF THE LUNG AND OF THE STOMACH AND OESOPHAGUS BY DIAZOMETHANE AND *N*-METHYL-*N*-NITROSO-URETHANE, RESPECTIVELY

REGINA SCHOENTAL AND P. N. MAGEE

*From the Toxicology Research Unit, Medical Research Council Laboratories, Woodmansterne Road, Carshalton, Surrey*

Received for publication February 1, 1962.

CONSIDERABLE evidence exists that many alkylating agents are cytotoxic and mutagenic and that some, notably the nitrogen mustards, diepoxides etc. also are weakly carcinogenic (Haddow, 1958). It appeared important, therefore, to test diazomethane, which is the simplest alkylating agent, and is known to be mutagenic (Rapoport, 1948). A methylating agent, possibly diazomethane, has also been postulated as an intermediary in the carcinogenic action of dimethylnitrosamine (Hultin *et al.*, 1960 ; Magee and Hultin, 1962 ; Magee and Farber, 1962 ; Emmelot and Mizrahi, 1961).

*N*-methyl-*N*-nitroso-urethane was also tested since it releases diazomethane in alkaline solutions, and at neutral pH when in contact with tissues and sulphydryl compounds (Schoental, 1961).

This paper describes the production of squamous carcinoma (and other lesions) of the lung (Fig. 1) by diazomethane and of the stomach (Fig. 2) and oesophagus (Fig. 3) by *N*-methyl-*N*-nitroso-urethane (NMU). A preliminary communication of some of these results has already been published (Schoental, 1960).

### MATERIALS AND METHODS

The experimental animals were rats and mice of both sexes. The white rats were either bred in this Laboratory from the Porton strain, or supplied by the Laboratory Animals Centre, Carshalton, from an inbred strain No. 606p, LAC catalogue of Uniform Strains, 2nd Ed. 1958. The mice of the A/2G strain were supplied by the Laboratory Animals Centre and Swiss albino mice were obtained from the National Institute for Medical Research, Mill Hill. All the animals were housed in metal cages and were given MRC Diet 41B and water *ad libitum*. The preparation of ethereal diazomethane from commercial *N*-methyl-*N*-nitroso-urethane, and the conditions of its application were the same as described in the preliminary communication (Schoental, 1960). The concentration of diazomethane in the ethereal solutions varied from 3·3–0·1 mg./ml. The solutions were kept at −15° C. in small conical flasks stoppered with polythene-covered glass stoppers.

The animals were exposed to inhalation twice a week by placing them in a desiccator, 6250 ml. capacity, in groups of 2–3 rats or 6 mice at a time. One ml. of ethereal solution of diazomethane, or of ether, was introduced from a syringe through a plastic sheet placed, instead of a stopper, over the opening of the desiccator's lid, and the animals were left in the respective atmosphere for the times specified in each experiment. When an animal appeared in poor condition, it was not subjected to treatment until it seemed sufficiently recovered.

For skin applications the ethereal solution of diazomethane was applied from a dropping pipette (2–3 drops at a time) to the clipped skin in the intrascapular region ; the mice were kept in the cage during the applications, which took place in a fume cupboard.

The rats were weighed at monthly intervals and all the animals were observed daily. Those animals which died, or which were killed by " coal " gas when found in poor condition, were autopsied and their lungs, livers, stomachs and some other organs were fixed in " Hellys " solution sectioned at 5 $\mu$ and stained routinely with haematoxylin and eosin for microscopic examination.

### EXPERIMENTAL

The following experiments were performed :

### *A. Diazomethane (DAM)*

(a) *Inhalation*

1. Seven white male rats (30–40 g. body weight) were exposed to DAM twice weekly for 6 months (2–3 minutes exposures) and two control rats were similarly exposed to inhalation of ether.
2. Six LAC 606p male rats (50–60 g. body weight) were exposed to DAM twice weekly for 4·5 months (1·5 minutes exposures), and two control rats were exposed similarly to inhalation of ether.
3. Twelve female A/2G mice were exposed once only to DAM for 5–6 minutes ; all died within 2 days after the exposure.
4. Twelve male A/2G mice were exposed to DAM for 3 minutes, the exposures were then reduced to 1–2 minutes when two of the animals died, and the treatment continued for 6 months. Twelve other male A/2G control mice were exposed in a similar way to ether.
5. Eight male Swiss mice were exposed to DAM for 5 months (1·5 minute exposures) and six male Swiss control mice were exposed to ether in a similar way.

(b) *Skin application*

Twelve A/2G male mice were treated with ethereal solutions of diazomethane five times weekly, the solutions (2–3 drops) were applied from a Pasteur pipette to the clipped skin at the intrascapular region without taking the mice from their box. This treatment, with occasional 2–3 weeks interruptions, was continued for 5 months.

(c) *Subcutaneous injections*

Ten male Swiss mice were given 8 monthly subcutaneous injections of 0·1 ml. of a solution of diazomethane in ether diluted (at the time of injection) with an equal volume of arachis oil. Four more monthly injections were made using undiluted ethereal solutions of diazomethane (0·1 ml. each).

### *B.* N-*Methyl*-N-*Nitroso-Urethane (NMU)*

1. Ten white male rats (100–125 g. body weight) were given by stomach tube 0·1 ml. of 50 per cent aqueous alcohol containing 20 mg. of NMU.

2. Six male white rats (95–120 g. body weight) were given in a similar way NMU in 5 per cent aqueous alcohol ; 3 rats received 5 mg./rat and 3 received 2·5 mg./rat.

3. Nine adult female and 3 adult male rats (170–205 g. body weight) and 3 male weanling rats (55–65 g. body weight) of the inbred LAC 606p strain were given by a short mouse-stomach tube 0·1 ml. of 50 per cent aqueous alcohol solution containing NMU, 5 mg./rat. These animals received 2·5 months later a second dose of NMU, 1 mg./rat.

4. Ten male and 5 female rats of the inbred LAC 606p strain, 40–80 g. body weight, were given 0·1 ml. of an aqueous-alcoholic solution of NMU by stomach tube, 2·5 mg./rat, followed 2·5 months later by a second dose, 2 mg./rat, and by a third dose, 1 mg./rat, two weeks after the second dose.

---

### EXPLANATION OF PLATES

Fig. 1.—Rat, male, diazomethane, 6 months. Killed 5 months later. Lung with squamous carcinoma.

Fig. 2.—Female LAC 606p rat, 2 doses *N*-methyl-*N*-nitroso-urethane. Killed 15·5 months after the second dose. Stomach with squamous carcinoma.

Fig. 3.—Female LAC 606p rat. 2 doses *N*-methyl-N-nitroso-urethane. Killed 11 months after the second dose. Squamous carcinoma of oesophagus.

Fig. 4.—Female LAC 606p rat, 2 doses *N*-methyl-*N*-nitroso-urethane. Killed 18·5 months after the second dose. Multiple tumours present in the stomach and in the oesophagus.

Fig. 5.—Female A/2G mouse, diazomethane inhalation. Killed 2 days after. H. and E. ×62. Acute perivascular, peribronchial and intra-alveolar oedema.

Fig. 6.—Male Swiss mouse, diazomethane inhalation 5 months. Killed 2 months later. Lung, areas of collapse fibroses with numerous pigment-laden macrophages. H. and E. ×62.

Fig. 7.—Male A/2G mouse, diazomethane inhalation 6 months. Killed 10 days later. Lung, area of squamous metaplasia. H. and E. ×62.

Fig. 8.—Same mouse as Fig. 6. Papillary ingrowth into the lumen of a tubular epithelial-lined structure, adjacent to adenoma. H. and E. ×62.

Fig. 9.—Male rat, diazomethane inhalation 6 months. Killed 4 months later. Pulmonary adenoma. H. and E. ×62.

Fig. 10.—Male rat, diazomethane, inhalation 6 months. Killed 5 months later. Squamous carcinoma of the lung. H. and E. ×62.

Fig. 11.—Rat same as Fig. 9. Metastic squamous carcinoma invading skeletal muscle. H. and E. ×62.

Fig. 12.—Swiss male mouse, diazomethane subcutaneous injection. Died 20·5 months, after the beginning of treatment. Spindle cell sarcoma invading skeletal muscle. H. and E. ×62.

Fig. 13.—Female rat, *N*-methyl-*N*-nitroso-urethane. Killed 48 hours. Acute destructive lesion of stomach with haemorrhage into mucosa and massive oedema of submucosa. H. and E. ×31.

Fig. 14.—Rat same as Fig. 13. Periportal necrosis of the liver. H. and E. ×62.

Fig. 15.—Female rat, *N*-methyl-*N*-nitroso-urethane. Killed after 7 days. Irregular regeneration of glandular mucosa and organisation of submucosa. H. and E. ×62.

Fig. 16.—Female LAC 606p rat ; 3 doses *N*-methyl-*N*-nitroso-urethane. Killed 13 months after the last dose. Hyperplasia of squamous epithelium at junction of squamous and glandular stomach and marked atrophy of adjacent squamous mucosa. H. and E. ×62.

Fig. 17.—Female LAC 606p rat, 2 doses *N*-methyl-*N*-nitroso-urethane. Killed 14 months after the second dose. Epithelial hyperplasia in glandular stomach with formation of irregular acini. H. and E. ×62.

Fig. 18.—Female LAC 606p rat, 2 doses *N*-methyl-N-nitroso-urethane. Killed 13·5 months after the second dose. Well differentiated squamous carcinoma of stomach with much keratin. H. and E. ×62.

Fig. 19.—The same tumour as in Fig. 17 under a higher magnification. H. and E. ×250.

Fig. 20.—Female LAC 606p rat, 2 doses *N*-methyl-N-nitroso-urethane. Killed 12·5 months after the second dose. Cross section of oesophageal squamous carcinoma. H. and E. ×2·6.

Fig. 21.—Female LAC 606p rat, 2 doses *N*-methyl-*N*-nitroso-urethane. Killed 11·0 months after second dose. Squamous carcinoma of oesophagus. H. and E. ×62.

Fig. 22.—Female LAC 606p rat, the same as in Fig. 4. Early tumour of the stomach. H. and E. ×33.

Schoental and Magee.

5

6

7

8

9

10

11

12

13

14

15

16

Schoental and Magee.

17

18

19

20

21

22

Schoental and Magee.

### RESULTS

*Diazomethane*

Table I summarises the experiments performed on rats and mice using diazomethane. For purposes of description these have been divided in 3 groups according to the time of survival of the animals.

TABLE I.—*Survival of Animals Treated with Diazomethane (DAM) and Ether*

| Animal species | Strain | Sex | Number | Treatment | Survival: Less than 10 days | Survival: Less than 10 months | Survival: More than 10 months |
|---|---|---|---|---|---|---|---|
| | | | | *Inhalation* 2 × *week* | | | |
| Rats | White | M. | 7 | DAM (2–3 min.) for 6 months | 1 | 4 | 2 |
| Rats | ,, | M. | 2 | Ether (2–3 min.) for 6 months | — | — | 2 |
| Rats | LAC 606p | M. | 6 | DAM (1·5 min.) for 4·5 months | 1 | — | 5 |
| Rats | ,, | M. | 2 | Ether (1·5 min.) for 4·5 months | — | — | 2 |
| Mice | A/2G | F. | 12 | DAM (5–7 min.) × 1 | 12 | — | — |
| Mice | ,, | M. | 12 | DAM (1–2 min.) for 6 months | 2 | 10 | — |
| Mice | ,, | M. | 12 | Control: Ether (2 min.) for 6 months | — | 8 | 4 |
| Mice | Swiss | M. | 8 | DAM (1·5 min.) for 5 months | 2 | 6 | — |
| Mice | ,, | M. | 6 | Control: Ether (1·5 min.) for 5 months | — | 2 | 4 |
| | | | | *Skin application* 5 × *week* | | | |
| Mice | A/2G | M. | 12 | DAM for 5 months | 2 | 6 | 4 |
| | | | | *Subcutaneous injections* 1 × *month* | | | |
| Mice | Swiss | M. | 10 | DAM for 12 months | 1 | — | 9 |

1. This group comprises animals which died in less than 10 days. These all had difficulty in breathing and were cyanosed. Post mortem examination showed severe pulmonary congestion and oedema (Fig. 5); other organs were not obviously involved apart from slight fatty change in the livers.

2. The second group includes animals which survived up to 10 months. In this group some died in the earlier months, shortly after the exposure, with acute lesions as described above, superimposed on more chronic changes.

The rats which survived after the treatment was stopped, all showed conspicuous lung injury. Typically, the lungs were voluminous and failed to collapse normally on opening the thorax. The lungs were often studded with whitish, hard nodules, sometimes involving all lobes, sometimes confined to parts. Congestion was marked in some regions. Microscopically, the lungs have conspicuously dilated bronchi, often surrounded by hypertrophied lymphoid zones with varying degrees of chronic inflammatory cellular infiltration. There are widespread areas of alveolar collapse with varying degrees of fibrosis and numerous pigment-laden macrophages (Fig. 6) and within these areas epithelial lined spaces are sometimes found. Squamous metaplasia is occasionally seen (Fig. 7). Patchy congestion is often present, sometimes severe, with haemorrhage into alveoli. Regions of compensatory emphysema are seen in the areas which are involved in the collapse fibrosis.

In the mice the changes are very similar, but especially with the Swiss mice the deposition of the iron-containing pigment is often much more marked, giving the whole organ a patchy brown colouration macroscopically. The hilar lymph glands were often enlarged.

The A/2G mice of this group had similar chronic pulmonary damage and in addition there were adenomata in 7 out of 10 survivors. Macroscopically the adenomata were multiple and of varying size up to 5 mm. in diameter. Microscopically, these were quite typical. Papillary ingrowth into the lumen of a tubular epithelial-lined structure is seen in one lung (Fig. 8). Some of the A/2G mice which had been treated with ether inhalation were killed at the time of death of the experimental mice. In this group two mice out of 8 had adenomata.

3. The third group contains animals surviving longer than 10 months. Among the 7 rats the chronic lung damage was more severe, and two animals had pulmonary adenomas, and another had adenoma and squamous carcinoma of the lung. The pulmonary adenomas appear to arise in areas of collapse fibrosis (Fig. 9). The rat with the squamous carcinoma of the lung was killed 11 months after the start of the experiment. Macroscopically the tumour consisted of hard white nodular masses involving much of both lungs (Fig. 1) and there were widespread deposits of similar character in the pleural cavity. Microscopically the tumour is a typical squamous carcinoma (Fig. 10) invading extensively in the lung, and the deposits have a similar structure. One of the metastatic nodules adherent to the diaphragm and invading the skeletal muscle is shown in Fig. 11.

Control rats exposed to inhalation of ether and killed at the same time as the last experimental animals had no significant lung changes.

All the Swiss mice given diazomethane by subcutaneous injection survived for longer than 1 year during which the treatment was given, until 26 months after the beginning of the experiment. All had chronic inflammatory changes in the lung and one had multiple pulmonary adenomas. One of these mice developed a large subcutaneous mass at the site of injection. Microscopically this was an actively growing spindle cell sarcoma invading muscle (Fig. 12). No abnormalities at the site of injection were seen in the other mice.

In the other organs studied no changes were found which could be attributed directly to the treatment.

*B.* N-*methyl*-N-*nitroso-urethane* (*NMU*)

Table II summarises the experiments performed on rats given NMU.

TABLE II.—*Survival of Animals Treated with* N-*Methyl*-N-*Nitroso-Urethane*

| Animal species | Strain | Sex | Number | Treatment | Survival: Less than 10 days | Survival: Less than 10 months | Survival: More than 10 months |
|---|---|---|---|---|---|---|---|
| Rats | White | M. | 10 | 20 mg. (orally) | 10 | — | — |
| Rats | ,, | M. | 3 | 5 mg. ,, | 1 | — | 2 |
| Rats | ,, | M. | 3 | 2·5 mg. ,, | — | 1 | 2 |
| Rats | LAC 606p | F. | 9 | (5+1) mg. ,, | — | 1 | 8 |
| Rats | ,, | M. | 6 | (5+1) mg. ,, | — | 3 | 3 |
| Rats | ,, | M. | 10 | (2·5+2+1) mg. ,, | 3 | 2 | 5 |
| Rats | ,, | F. | 5 | (2·5+2+1) mg. ,, | — | — | 5 |

1. The 10 rats given NMU, 20 mg./rat, by stomach tube all died within 3 days. They developed subcutaneous oedema, and various degrees of pulmonary congestion and oedema. The livers were pale, mottled and friable, with a distinct pattern of lobulation. The squamous part of the stomach was dark red, with haemorrhage into the wall. On opening the stomach there was a severe lesion of the mucosa which was covered by mucinous slough.

Microscopically, there is severe damage to the squamous part of the stomach with rather less marked injury to the glandular part, becoming less severe as the pylorus is approached. The duodenum appears to escape. The whole thickness of the wall of the squamous stomach may be destroyed down to the serous layer, with massive haemorrhage into the necrotic tissue. At the junction with squamous, the glandular part may be equally severely damaged, but more commonly there is intense vascular engorgement of the mucosa with some necrosis and marked oedema of the submucosa (Fig. 13). Moving towards the pylorus the mucosal damage becomes less severe, but the submucosal oedema persists so that the mucosa is widely separated from the muscle layers.

In the earlier stages of the lesion there is little cellular reaction, but later there is often an intense invasion of the oedematous submucosa by eosinophils followed by fibroblastic activity and organisation. In many of these rats there is a zonal periportal necrosis of the liver involving all lobules (Fig. 14). This necrosis is followed by parenchymal cell regeneration accompanied by intense mitotic activity.

With smaller doses of NMU (5 mg. or less) the initial stomach lesion was of a similar character but less severe and the majority of the rats recovered. Microscopically a week after the dose there is considerable degree of organisation and repair in the submucosa and regeneration of the glandular epithelium. This is sometimes irregular with formation of gland-like spaces (Fig. 15). In the grossly damaged squamous part, there may be little epithelial regeneration over the layer of grossly damaged stomach wall which is undergoing organisation and repair. As recovery from the initial damage progresses there is macroscopically obvious reduction in the amount of squamous stomach present, and microscopically it is now seen to consist mainly of a fibrous layer which separates the regenerated squamous epithelium, some of which may be greatly flattened (Fig. 16), from what remains of the muscle layers and the serosa. At the junction with the glandular stomach there is often a region of squamous epithelial hyperplasia and on the glandular side, less commonly, there may also be hyperplasia with formation of irregular epithelial lined acinar structures in the mucosal wall (Fig. 17).

The squamous epithelial hyperplasias may be early stages in the development of carcinomas, but no tumours of the glandular stomach have been observed.

Among 14 rats surviving 16 months or longer 4 developed tumours of the stomach. One of these was an enormous mass replacing most of the stomach and adherent to spleen and intestines. There were numerous whitish small nodules, throughout the peritoneal cavity. There was also a whitish hard tissue mass in the left lung. Microscopically the stomach lesion is a squamous carcinoma and the peritoneal nodules have the same structure. Unfortunately, the post mortem changes prevented confident histological diagnosis of the lung mass.

Fig. 2 shows the macroscopic appearance of another stomach tumour. The lesion was a hard whitish nodular mass occupying a large part of the stomach, which shows the characteristic reduction in size of the squamous part. There were no metastases. Microscopically, the tumour is a typical well differentiated squamous carcinoma with abundant keratin formation (Fig. 18 and 19).

The third tumour was a small hard nodule in the stomach wall about 5 mm. in diameter, which had a similar microscopic structure.

In other animals of this series, as well as tumours of the stomach, there occurred 5 oesophageal carcinomas. One of these is shown in Fig. 3. The lesion was a hard whitish annular mass in the middle part of the oesophagus causing great thickening of the wall (Fig. 20). The other tumours were essentially similar, two being larger. Microscopically they are all typical squamous carcinomas (Fig. 21) indistinguishable from those of the stomach. These tumours are locally invasive, extending close to the trachea and great vessels, but not invading them.

One other female in this series, killed when in poor condition 19 months after the first dose of NMU, had multiple tumours. These formed several whitish nodules in the lower part of the oesophagus and in the stomach, the squamous part of which was shortened (Fig. 4 and 22). This animal had also a pituitary tumour, but such tumours have been seen among untreated old females of our rat colony.

No liver lesions were seen in rats which survived longer than a few days after NMU.

In experiments in which rats were given subcutaneous injections of NMU, 25 mg./rat, the animals died after 24 to 48 hours. Macroscopically the lungs were intensely and uniformly engorged and had a dark reddish-purple colour. Microscopically there is generalised severe congestion and oedema, the appearance being quite similar to that produced acutely by diazomethane. At the site of injection there is local tissue destruction with intense congestion and haemorrhage.

### DISCUSSION

The experiments described here were of an exploratory nature, undertaken with a view to testing the carcinogenic activity of simple alkylating agents. Because of their acute toxic effects dosage and mode of application of these agents had often to be modified, especially in the course of experiments with diazomethane. Whether the compound was applied to the skin, by subcutaneous injection or by inhalation lung lesions commonly developed in both rats and mice. In A/2G mice, in which lung adenomata develop in old age, the incidence of these tumours was greatly accelerated and increased. Thus the eight mice which survived skin applications of ethereal diazomethane solutions for 5 months all had adenomata, often multiple, when they died in the course of the following 7 months. Among control A/2G mice exposed to ether inhalation for a similar period, 2/8 mice had incipient lung adenomata when killed at a comparable time.

All the Swiss mice exposed to inhalations of diazomethane for 6 months died in the course of the following 1·5 months. They showed severe lung lesions but no distinct tumours were seen.

In the group of Swiss mice given subcutaneous injections of diazomethane monthly, the survival was very good, up to 26·5 months. All mice had chronic lung lesions and one of the mice surviving longest had adenomas in the lung; here the dosage of diazomethane solutions in ether or in a mixture of ether and arachis oil had to be very small (less than 0·1 mg./dose) in order to avoid excessive local tissue damage. It is uncertain whether the sarcoma which developed at the site of injection can, in this series, be attributed to the interaction of diazomethane with subcutaneous tissue or to a product formed by its action with arachis oil.

White rats are prone to chronic inflammatory lung disease ; many of the histological appearances seen in the lungs of our experimental animals at various stages cannot be distinguished from such inflammatory conditions. Pulmonary adenomata, however, are very rare in rats and have not been observed in our colony. The fact that among the small numbers of rats treated with diazomethane several developed tumours and one had a squamous carcinoma, suggests that these were due to the treatment. Neither the control rats exposed to inhalation of ether nor rats used in other experiments showed such lesions.

Diazomethane is very reactive and will form a variety of products when it interacts with tissue constituents. Not all of these products are likely to be involved in its carcinogenic action. Multiple applications are necessary for the induction of lung tumours. No epithelial tumours were formed in mice at the site of DAM application to the skin possibly because it interacted with keratin, which might have protected the deeper tissue layers. The main part of the applied DAM probably evaporated, was inhaled by the mice in which it induced lung adenomas.

Diazomethane is known to be a very irritating agent and this is a complicating factor in considering its purely carcinogenic action. The role of chronic irritation has been largely abandoned (Berenblum, 1944) and it is improbable, though it cannot be excluded, that its irritating action contributed to the development of tumours. This irritating and necrogenic action of diazomethane also restricts the dose per application ; the margin between effective and lethal dose appears to be narrow.

It has been reported recently that continuous exposure to *N*-methyl-*N*-nitrosourethane given in the drinking water throughout the lifespan gave rise to stomach tumours in rats (Druckrey, *et al.*, 1961). Our results indicate that one or two doses only of NMU are sufficient to induce squamous carcinomas of the stomach and oesophagus, which only become apparent months later. The oesophageal tumours which appeared after the use of the short administration tube presumably arose at the site of application of the compound, since NMU evidently interacts immediately with the tissues. From experiments *in vitro*, it has been shown that NMU decomposes very rapidly in contact with tissues, probably releasing diazomethane (Schoental, 1961). The mechanism of action of both these compounds, diazomethane and NMU is therefore probably the same. However, the fact the NMU is effective with even a single dose may be related to its decomposition at neutral pH by sulphydryl compounds (Schoental, 1961) which may react with evolved diazomethane and thus be involved in the carcinogenic process.

Recent work on dimethylnitrosamine has suggested that alkyl nitrosamines are carcinogenic by being transforced enzymatically into alkylating agents (Hultin *et al.*, 1960 ; Magee and Hultin, 1962 ; Magee and Farber, 1962 ; Emmelot and Mizrahi, 1961). Thus their mode of action could be similar to that of diazomethane and NMU. Dimethylnitrosamine induces in rats liver (Magee and Barnes, 1956), kidney (Magee and Barnes, 1959 ; Schmahl and Preussmann, 1959) and lung tumours (Zak *et al.*, 1960 ; Argus and Hoch-Ligeti, 1961). It remains, however, to be shown which of the products of interaction between the alkylating agents and tissue constituents are responsible for the development of tumours.

The remarkable feature of the experiments with NMU is that cancers were induced by one or two doses of this substance which is water soluble at the concentration used at physiological pH. In this respect stomach and oesophageal cancers can be added to liver and kidney tumours, which have been shown to develop after a single dose of the carcinogens, pyrrolizidine alkaloids (Schoental, unpublished results) and dimethylnitrosamine respectively (Magee and Barnes, 1962). Evidently the critical cellular change occurs very soon after the carcinogen reacts with the appropriate cell constituents and continues irrevocably, occasionally erupting into cancer without any apparent additional specific treatment. To say that this change resembles somatic mutation does not explain the underlying biochemical changes which are equally obscure in mutations as they are in carcinogenesis.

The results reported here may have some bearing on the development of corresponding tumours in man. Alkyl radicals are likely to be formed during the burning of organic matter (in fried food, or smoking tobacco, etc.)

### SUMMARY

1. White rats exposed to inhalation of diazomethane developed various acute and chronic lung lesions, and also adenoma and squamous lung carcinoma.

2. In mice diazomethane induced similar acute and chronic lung lesions and lung adenoma regardless of the route of its administration ; inhalation, subcutaneous injection or skin application.

3. One mouse developed a subcutaneous spindle cell sarcoma at the site of injection of solutions of diazomethane.

4. Oral administration of *N*-methyl-*N*-nitroso-urethane to rats induced acute and chronic lesions of the stomach and also squamous carcinomas of the stomach and of the oesophagus, which appeared more than a year after one, two or three doses of this compound.

5. The bearing of these results on the alkylating hypothesis of carcinogenesis is discussed.

We wish to thank Dr. J. M. Barnes for his interest and help, Mr. R. F. Legg for the microphotographs and Miss A. Mackinnon and Mr. M. R. Greenwood for valuable technical assistance.

### REFERENCES

Argus, M. F. and Hoch-Ligeti, C.—(1961) *J. nat. Cancer Inst.*, **27**, 695.
Berenblum, I.—(1944) *Arch. Path.*, **38**, 233.
Druckrey, H., Preussmann, R., Schmähl, D. and Müller, M.—(1961) *Naturwissenschaften*, **48**, 165.
Emmelot, P. and Mizrahi, I. J.—(1961) *Nature, Lond.*, **42**, 192.
Haddow, A.—(1958) *Brit. med. Bull.*, **14**, 79.
Hultin, T., Arrhenius, E., Löw, H. and Magee, P. N.—(1960) *Biochem J.*, **76**, 109.
Magee, P. N. and Barnes, J. M.—(1956) *Brit. J. Cancer*, **10**, 114.—(1959) *Acta Un. int. Cancr.*, **15**, 187.—(1962) *J. Path. Bact.* (in press).
*Idem* and Farber, E.—(1962) *Biochem. J.*, **83**, 114.
*Idem* and Hultin, T.—(1962) *Ibid.*, **83**, 106.
Rapoport, I. A.—(1948) *Dokl. Akad. Nauk, S.S.S.R.*, **59**, 1183.
Schoental, R.—(1960) *Nature, Lond.*, **188**, 420.—(1961) *Ibid.*, **192**, 670.
Schmähl, D. and Preussmann, R.—(1959) *Naturwissenschaften*, **46**, 175.
Zak, F. G., Holzner, J. H., Singer, E. J. and Popper, H.—(1960) *Cancer Res.*, **20**, 96.